# The Mechanism of the Photostability Enhancement of Thin-Film Transistors Based on Solution-Processed Oxide Semiconductors Doped with Tetravalent Lanthanides

**DOI:** 10.3390/nano12213902

**Published:** 2022-11-04

**Authors:** Linfeng Lan, Chunchun Ding, Penghui He, Huimin Su, Bo Huang, Jintao Xu, Shuguang Zhang, Junbiao Peng

**Affiliations:** 1 State Key Laboratory of Luminescent Materials and Devices, South China University of Technology, Guangzhou 510640, China; 2 School of Physics and Electronics, Hunan University, Changsha 410082, China

**Keywords:** praseodymium, terbium, tetravalent, oxide semiconductors, thin-film transistors, stability

## Abstract

The applications of thin-film transistors (TFTs) based on oxide semiconductors are limited due to instability under negative bias illumination stress (NBIS). Here, we report TFTs based on solution-processed In_2_O_3_ semiconductors doped with Pr^4+^ or Tb^4+^, which can effectively improve the NBIS stability. The differences between the Pr^4+^-doped In_2_O_3_ (Pr:In_2_O_3_) and Tb^4+^-doped In_2_O_3_ (Tb:In_2_O_3_) are investigated in detail. The undoped In_2_O_3_ TFTs with different annealing temperatures exhibit poor NBIS stability with serious turn-on voltage shift (Δ*V_on_*). After doping with Pr^4+^/Tb^4+^, the TFTs show greatly improved NBIS stability. As the annealing temperature increases, the Pr:In_2_O_3_ TFTs have poorer NBIS stability (Δ*V_on_* are −3.2, −4.8, and −4.8 V for annealing temperature of 300, 350, and 400 °C, respectively), while the Tb:In_2_O_3_ TFTs have better NBIS stability (Δ*V_on_* are −3.6, −3.6, and −1.2 V for annealing temperature of 300, 350, and 400 ℃, respectively). Further studies reveal that the improvement of the NBIS stability of the Pr^4+^/Tb^4+^:In_2_O_3_ TFTs is attributed to the absorption of the illuminated light by the Pr/Tb4*f* ^n^—O2*p*^6^ to Pr/Tb 4*f* ^n+1^—O2*p*^5^ charge transfer (CT) transition and downconversion of the light to nonradiative transition with a relatively short relaxation time compared to the ionization process of the oxygen vacancies. The higher NBIS stability of Tb:In_2_O_3_ TFTs compared to Pr:In_2_O_3_ TFTs is ascribed to the smaller ion radius of Tb^4+^ and the lower energy level of Tb 4*f* ^7^ with a isotropic half-full configuration compared to that of Pr 4*f* ^1^, which would make it easier for the Tb^4+^ to absorb the visible light than the Pr^4+^.

## 1. Introduction

Oxide semiconductors, such as InGaZnO_4_ (IGZO) [1,2,3,4,5], have drawn considerable attention for the advantages of relatively high mobility, large-area processability, good uniformity, high transparency to visible light, etc. [6] In particular, the extremely low off current (*I_off_*) makes it attractive in energy-saving devices that require long stand-by time [7]. However, a critical technical issue remains to be solved for better device applications of the thin-film transistors (TFTs) based on oxide semiconductors. Although oxide semiconductors are highly transparent in the visible range, they suffer from serious threshold voltage (*V_th_*) change under negative bias illumination stress (NBIS) even when illuminated by visible light with smaller photon energies than their bandgaps [8,9,10,11,12]. Although the NBIS instability of the oxide TFTs have been studied intensively over the past decade, no consensuses have been reached on the mechanism of the NBIS instability [6].

Our previous work shows that the doping of tetravalent lanthanides (Ln)—praseodymium (Pr^4+^) and terbium (Tb^4+^)—can improve the NBIS stability of the oxide TFTs greatly [13]. However, the insightful effects of the tetravalent lanthanides on photostability of the oxide TFTs is still unclear. In this paper, the differences between Pr^4+^ and Tb^4+^ doped oxide semiconductors are compared in detail, and new experiments and analysis (such as low-temperature measurements) are carried out to give a more insightful understanding of the mechanism of the NBIS instability of oxide TFTs and the intrinsic effect of Pr^4+^/Tb^4+^ on the electrical and optical properties of oxide TFTs.

## 2. Experimental Section

### 2.1. Precursor Solutions

The oxide semiconductor films were deposited by spin-coating and thermal decomposition of precursor solutions. A 0.2 M In_2_O_3_ solution was prepared by dissolving indium nitrate hydrate ((In(NO)_3_·nH_2_O), Sigma-Aldrich, Tianhe District, Guangzhou, China) in deionized water. The Ln:In_2_O_3_ precursor solutions were synthesized by dissolving indium nitrate hydrate ((In(NO)_3_·nH_2_O), Sigma-Aldrich, Tianhe District, Guangzhou, China) and lanthanides nitrate hydrate (Pr/Tb(NO)_3_·nH_2_O, Aladdin, Industrial Co., Shanghai, China) in deionized water, which was optimized to the total concentration of metal ion of 0.2 M and In/Ln molar ratio of 19:1. All the precursor solutions were stirred vigorously for 12 h at room temperature and filtered through a 0.22 μm syringe filter before spin-coating.

### 2.2. Device Fabrication

**Figure 1:**
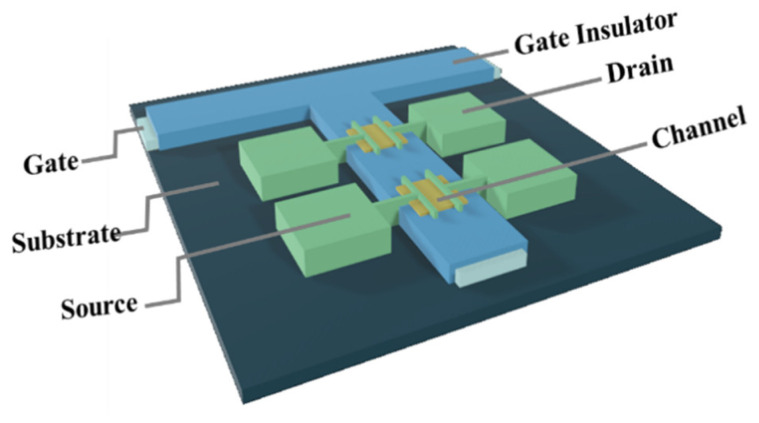
Schematic structure of the TFTs with an anodized gate dielectric.

A bottom-gate and top-contact structure was used to fabricate Ln:In_2_O_3_ TFT, as shown in Figure 1. First, a 300-nm-thick Al:Nd alloy film was deposited onto a glass substrate by sputtering and patterned by wet etch, followed by an anodization process to form a 200-nm-thickness Nd:AlO_x_ gate dielectric layer on the surface of Al:Nd film [14]. Next, an ultraviolet light irradiating for a long time was used to treat a part (channel area) of the Nd:AlO_x_ surface with a stencil shadow mask in order to form a hydrophilic surface in the channel area. The Ln:In_2_O_3_ precursor films were deposited onto the wettable area irradiated by UV by spin-coating the precursor solutions at 2000 rpm for 5 s and 6500 rpm for 40 s, followed by drying at 40 °C and thermal annealing at 300/350/400 °C for 1 h in an air condition. Then, the Al source and drain electrodes were deposited on the Ln: In_2_O_3_ layer by thermal evaporation and defined the channel area with 800 μm width (*W*) and 200 μm length (*L*) by using a stencil shadow mask. Finally, the devices were post-annealed at 300 °C for 1 h in an air condition.

### 2.3. Characterization of Films and Devices

The electrical characteristics of the TFTs were measured using a semiconductor parameter analyzer system in conjunction with a probe station in a vacuum condition. The NBIS stability was tested by monitoring evolutions of the transfer curves of the TFTs as a function of the stress time under gate bias stresses of −20 V combined with white LED irradiation (250 Lux). The crystallization characteristics of the films were determined by X-ray diffraction (XRD) experiments. The chemical shift of different elements was characterized by the X-ray Photoelectron Spectroscopy (XPS).

## 3. Results and Discussion

### 3.1. TFT Characteristics

**Figure 2:**
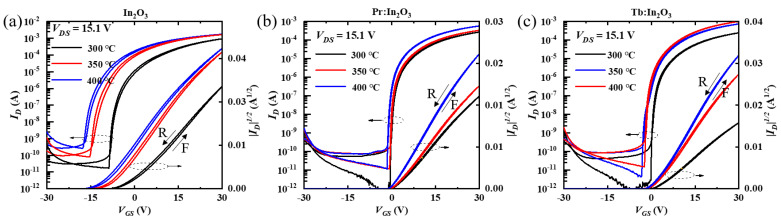
Transfer curves of the (**a**) In_2_O_3_, (**b**) Pr:In_2_O_3_ TFTs, and (**c**) Tb:In_2_O_3_ TFTs; all of the curves recorded both forward (F) and reverse (R) gate sweeps.

The transfer curves of the In_2_O_3_, Pr:In_2_O_3_, and Tb:In_2_O_3_ TFTs with different annealing temperature are shown in Figure 2a–c, respectively. All the TFTs showed increases on the current (*I_on_*) and off current (*I_off_*) with increasing annealing temperatures, which reflects that the channels became more conductive as the annealing temperature increased. Compared to those of the Pr:In_2_O_3_ and Tb:In_2_O_3_ TFTs, the In_2_O_3_ TFTs displayed higher *I_off_*, more negative *V_th_*, and more sensitivity to the annealing temperature, which reflects that both Pr and Tb can reduce the free carriers. The saturation mobility (*μ_sat_*) of TFTs were calculated using Equation (1).

The properties of the In_2_O_3_, Pr:In_2_O_3_, and Tb:In_2_O_3_ TFTs are summarized in Table 1. Although the In_2_O_3_ TFTs had higher mobility, they exhibited large hysteresis in the transfer curves between forward and reverse gate sweeps, which suggests that the In_2_O_3_ TFTs were in a rather unstable state even annealed at 400 °C. For Pr:In_2_O_3_ TFTs, the mobility increased slightly from 5.0 to 6.1 cm^2^·V^−1^·s^−1^ as the annealing temperature increased from 300 °C to 350 °C; when the annealing temperature further increased to 400 °C, the mobility increased largely to 10.1 cm^2^·V^−1^·s^−1^. For Tb:In_2_O_3_ TFTs, the mobility at 300 °C annealing temperature was only 4.7 cm^2^·V^−1^·s^−1^, which was a bit lower than that of the Pr:In_2_O_3_ TFTs. As the annealing temperature increased to 350 °C, the mobility increased significantly to 13.4 cm^2^·V^−1^·s^−1^, which was much higher than that of the Pr:In_2_O_3_ TFTs. When the annealing temperature further increased to 400 °C, the mobility further increased to as high as 18.2 cm^2^·V^−1^·s^−1^. It can be briefly summarized that, at a low annealing temperature (300 °C), the Pr:In_2_O_3_ TFT and Tb:In_2_O_3_ TFT showed little difference on mobility, but at high annealing temperature (350/400 °C), the Tb:In_2_O_3_ TFTs had much higher mobilities than the Pr:In_2_O_3_ TFTs.

### 3.2. Film Structures

As known, in solution-processed oxide semiconductors, increasing annealing temperature is good for reducing the chemical residues and hence increasing the mobility, which is consistent with the results summarized in Table 1 [15]. For TFTs with the same annealing temperature (350 or 400 °C), the higher mobility of the Tb:In_2_O_3_ TFTs compared to that of the Pr:In_2_O_3_ TFTs may be attributed to the smaller ions radius of the Tb ions compared to the corresponding Pr ions. Unlike most of the other elements, lanthanides with larger atomic numbers have smaller ion radii due to the lanthanide contradiction, so Tb has a smaller ion radius (0.92 Å for Tb^3+^, and 0.76 Å for Tb^4+^) than Pr (0.99 Å for Pr^3+^, and 0.85 Å for Pr^4+^). Therefore, the ion radius of Tb is closer to that of In^3+^ (0.80 Å) compared to those of Pr, which may result in being easier to incorporate into the In_2_O_3_ lattice of Tb than Pr. The structures of the solution-processed Pr:In_2_O_3_ and Tb:In_2_O_3_ films annealed at different temperature were characterized by XRD experiments, as shown in Figure S1. All of the films showed a relatively strong diffraction peak around 30.6°, which is close to the (222) of the bixbyite In_2_O_3_, and a weak diffraction peak around 35.2°, which is close to the (400) of the bixbyite In_2_O_3_. Both Pr:In_2_O_3_ and Tb:In_2_O_3_ films exhibited better crystallinity as the annealing temperature increased. The 300 °C-annealing Tb:In_2_O_3_ film exhibited a much stronger (222) diffraction peak than the 300 °C-annealing Pr:In_2_O_3_ one. For the films annealed at higher temperatures (350 and 400 °C), there were no clear differences in the (222) diffraction peaks between Pr:In_2_O_3_ and Tb:In_2_O_3_, but the Tb:In_2_O_3_ showed a stronger (400) peak than the Pr:In_2_O_3_. Interestingly, the diffraction peak of the Pr:In_2_O_3_ film was almost in the same position as that of In_2_O_3_ film, while the diffraction peak of the Tb:In_2_O_3_ film exhibited an apparent left shit (~0.2°) compared to the standard (222) diffraction peak of the pure In_2_O_3_. The result implied that the Tb ions (at least some of them) may incorporate into the In_2_O_3_ lattice (causing lattice expansion) while most of the Pr ions may not incorporate into the In_2_O_3_ lattice (still In_2_O_3_ phase after Pr doping). Compared to the Pr:In_2_O_3_ with separated phases of PrO_x_ and In_2_O_3_, the Tb:In_2_O_3_ with Tb incorporated into the In_2_O_3_ lattice is better for carrier transport, which may result in higher mobility.

### 3.3. NBIS Instabilities

**Figure 3:**
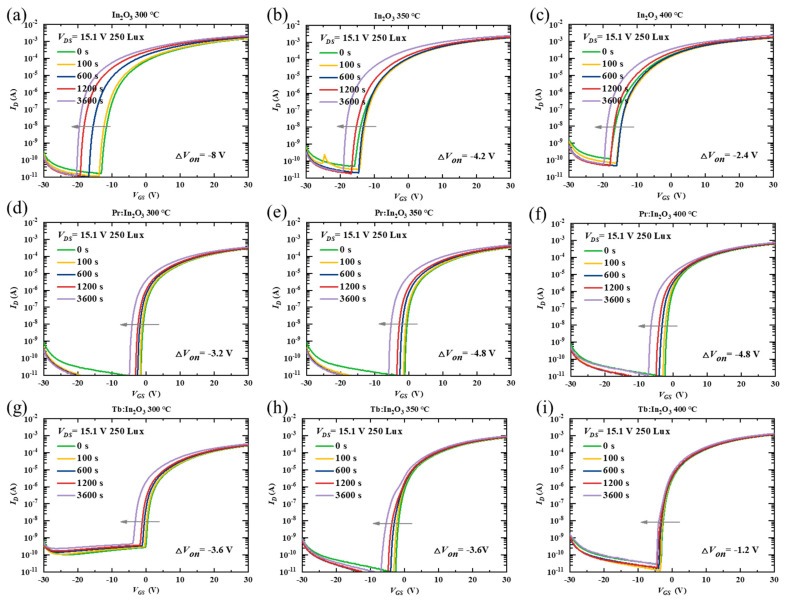
Variations of time-dependent transfer curves under NBIS (a negative gate bias stress of −20 V combined with white LED light illumination of 250 Lux) for the TFTs with channels of In_2_O_3_ (annealed at (**a**) 300 °C, (**b**) 350 °C, (**c**) 400 °C), Pr:In_2_O_3_ (annealed at (**d**) 300 °C, (**e**) 350 °C, (**f**) 400 °C), and Tb:In_2_O_3_ (annealed at (**g**) 300 °C, (**h**) 350 °C, (**i**) 400 °C).

To further investigate the effect of Pr/Tb on the TFT properties, the stability of the TFTs were characterized under NBIS with a negative *V_G_* stress of −20 V combined with an illumination stress of 250-Lux white LED light. Figure 3 shows the transfer curve evolutions of different TFTs as a function of the stress time during NBIS. The *V_on_* shift (Δ*V_on_*, defined by the gate voltage shift at *I_D_* = 10^−8^ A) under 3600 s NBIS are summarized in Table 1. The In_2_O_3_ TFTs with different annealing temperatures exhibited poor NBIS stability with large Δ*V_on_* (the actual Δ*V_on_* of the In_2_O_3_ TFTs was much larger than the measured Δ*V_on_*, as discussed below). After doping with Pr/Tb, the TFTs showed greatly improved NBIS stability. Surprisingly, the Pr:In_2_O_3_ and the Tb:In_2_O_3_ TFTs exhibited different temperature-dependent trends. As the annealing temperature increased, the Pr:In_2_O_3_ TFTs had poorer NBIS stability (Δ*V_on_* were −3.2, −4.8, and −4.8 V for annealing temperature of 300, 350, and 400 °C, respectively), while the Tb:In_2_O_3_ TFTs had better NBIS stability (Δ*V_on_* were −3.6, −3.6, and −1.2 V for annealing temperatures of 300, 350, and 400 ℃, respectively).

It should be noted that the *V_on_* of all the undoped-In_2_O_3_ TFTs with different annealing temperatures reached the negative limit of −20 V (the voltage of the stressing gate bias) after 3600 s NBIS, which means that the actual Δ*V_on_* of the undoped-In_2_O_3_ TFTs was much larger than the measured Δ*V_on_*. To confirm the dependence of the *V_on_* shift limit on the stressing gate bias, the TFTs were characterized under NBIS with a more negative stressing gate bias of −30 V combined with an illumination stress of 250-Lux white LED light. Figure S2 shows the evolutions of the transfer curves of different TFTs under −30 V NBIS as a function of the stress time. The Δ*V_on_* of the In_2_O_3_ TFTs under −30 V NBIS were much larger than those under −20 V NBIS, and the negative limit of the *V_on_* of the In_2_O_3_ TFTs after NBIS is close to −30 V. Thus, the NBIS instability of the In_2_O_3_ TFTs were much more serious than measured. In contrast to the undoped In_2_O_3_ TFTs, the Pr/Tb:In_2_O_3_ TFTs did not show much difference for Δ*V_on_* under −30 V NBIS, as shown in Figure S3.

### 3.4. Oxygen Vacancies

**Figure 4:**
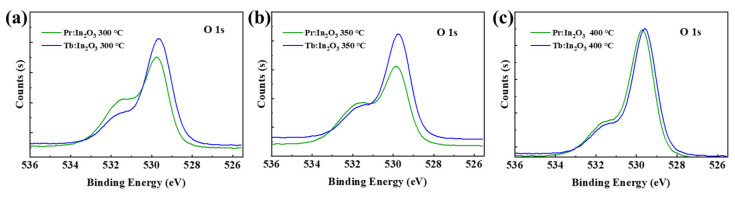
O 1s XPS spectra of Pr/Tb:In_2_O_3_ annealed at (**a**) 300, (**b**) 350, and (**c**) 400 °C.

It has been reported that the decrease in V_O_ concentration of the IGZO can improve the NBIS stability effectively [16]. Compared to the IGZO, the In_2_O_3_ has much higher V_O_ concentration. Although the lanthanide doping can reduce the V_O_ concentration and suppress the free carrier generation of the In_2_O_3_, the V_O_ concentration is still much higher than that of the IGZO. Here, the large improvement of NBIS stability after Pr/Tb doping is not mainly due to the reduction of the V_O_ concentration, because there are no direct relationships between the NBIS stability and the V_O_ concentration [13,17,18]. For example, the Pr:In_2_O_3_ had much higher V_O_ concentration than Tb:In_2_O_3_ (see Figure 4) for annealing temperature of 300 ℃, but it did not exhibit much difference in NBIS stability compared with Tb:In_2_O_3_. The In_2_O_3_ doped with another lanthanide element gadolinium (Gd, which has low electronegativity and large metal–oxide bonding energy that is comparable to those of Pr and Tb) had a low V_O_ concentration, but the Gd:In_2_O_3_ TFT had poor NBIS stability with Δ*V_on_* of as large as −13.9 V [13] for Pr:In_2_O_3_ annealed at 400 °C, the V_O_ concentration reduced greatly compared to the one annealed at 300 °C, but its NBIS stability was not as good as the one annealed at 300 °C. Thus, the reduction of V_O_ concentration is not the main reason for the large improvement of NBIS stability after Pr/Tb doping.

### 3.5. Temperature-Dependent Performances

**Figure 5:**
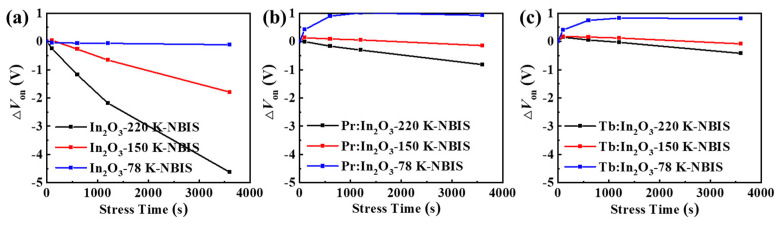
Δ*V_on_* evolutions of the TFTs with (**a**) pure In_2_O_3_, (**b**) Pr:In_2_O_3_, and (**c**) Tb:In_2_O_3_ under NBIS with different temperatures of 78 K, 150 K, and 220 K.

**Figure 6:**
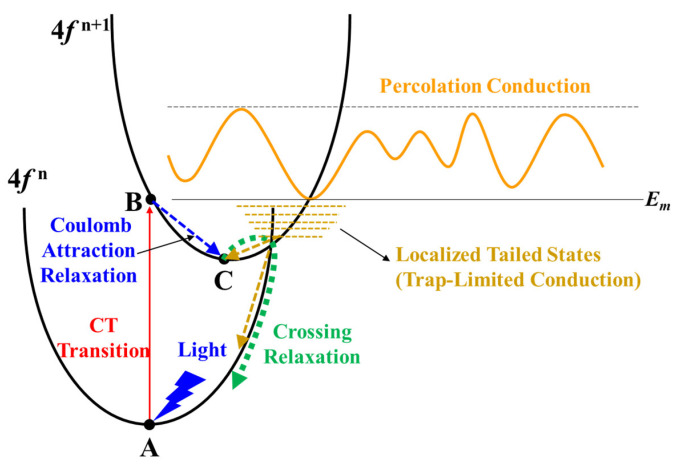
Illustration of the CT transition process in Pr^4+^/Tb^4+^ doped In_2_O_3_.

To further investigate the intrinsic mechanism of the NBIS stability, the NBIS stability measurements were performed at different temperatures (from 78 K to 295 K). Figure 5 shows the Δ*V_on_* evolution of the different TFTs under NBIS, and Figure S4 shows the corresponding evolutions of the transfer curves of different TFTs under NBIS. All of the TFTs showed decreasing *I_on_* with decreasing measuring temperature, which implies that trap-limited conduction (TLC) becomes dominant at a low temperature. In oxide semiconductors, both TLC and percolation conduction (PC) exist (see Figure 6) [19,20]. In TLC, the electrons are subjected to multiple trapping events in the localized tail states below the conduction band edge (*E_m_*), and the transport between tail states is via the variable range hopping (VRH) [20]. At this low temperature, the electrons are more likely to be trapped in the localized tail states due to the lack of sufficient thermal energy, thus the mobility decreases as the measuring temperature decreases. As a result, the conductivity of the oxide semiconductors reduces greatly at a low temperature, which is consistent with the reduction of the *I_on_* of the TFTs at low temperatures (see Figure S4).

It could also be seen from Figure 5 and Figure S4 that all devices displayed decreasing Δ*V_on_* with decreasing temperature. Surprisingly, when the measuring temperature decreased to 78 K, the pure In_2_O_3_ TFT showed little Δ*V_on_* after 3600 s NBIS, while the Pr:In_2_O_3_ and Tb:In_2_O_3_ TFTs exhibited positive Δ*V_on_* of +1.0 V and +0.8 V, respectively. For comparison, the stability under negative bias stress (NBS) without light illumination were tested at room temperature, as shown in Figure S5. Only the pure In_2_O_3_ TFTs showed obvious Δ*V_on_* of −3.2 V after 3600 s NBS at room temperature (while the Pr/Tb:In_2_O_3_ TFTs exhibited neglectable Δ*V_on_*). The results reflect that the NBIS instability is a combined effect of light illumination and temperature (thermal activation), as well as gate bias. The energy of the white LED light (or blue light) is not enough to activate the deep donates (such as V_O_) near valance band maximum (VBM) at low temperature (78 K), which is contrary to the concept that the activation energy of the subgap state formed by V_O_ is lower than the energy of the blue LED light.

### 3.6. Mechanisms of NBIS Instability

Over the past decades, considerable research efforts have been made to understand the native defects/impurities present in oxide semiconductors, but the origin of the unintentional *n*-type conductivity and/or the NBIS instability of the oxide TFTs is still a subject of debate [21,22,23,24,25,26,27,28,29,30]. Specifically, defect formation and carrier generation, electron localization/transient behavior, as well as carrier scattering in an amorphous state are all far from being fully understood. However, there seems to be an undeclared consensus that the V_O_ is the major defect in *n*-type oxide semiconductors. Very recent studies show that V_O_ likely acts as both shallow and deep donors in In_2_O_3_ [21,22]. The localized valence tail states or the subgap states near VBM, which are determined not only by their density but also by the degree of localization (structural defects in the amorphous state), contribute to the optical absorption within the visible range, i.e., from 1.5 eV to 3 eV [17]. Under combined stress of light illumination, temperature, and negative gate bias, some of the V_O_s will be excited, delocalized free electrons to the conduction band [23,24,25,26,27]. The transition of the V_O_ ground state to singly charged oxygen vacancy (Vo^1+^) or doubly charged oxygen vacancy (Vo^2+^) excited states causes spontaneous outward relaxation. The relaxation for V_O_ to Vo^2+^ is strong because of the electrostatic repulsion of the two positive charges in Vo^2+^ (e.g., the four nearest In neighbors of the Vo^2+^ in In_2_O_3_ significantly relax outward by 9.3%, 9.6%, 7.7%, and 13%) [21]. The strong relaxation causes a very slow V_O_ ionization/recombination process, which leads to a very long decade time of the photocurrent [28,29]. However, since the highest energy of the white LED light (the blue light, ~2.7 eV) is high enough for exciting some of the less-deep V_O_ states directly at low temperatures, the V_O_ ionization model cannot fully explain why the In_2_O_3_ TFTs exhibited little Δ*V_on_* at a low temperature (78 K).

Flewitt and Powell proposed a defect transition model to interpretate why the Δ*V_on_* is the combined effect of light illumination, temperature, and negative gate bias. According to the model, the electron donor defect (D_e_) can transfer to the neighboring n-coordinated oxygen atom [O(―M)_n_] via oxygen interstitials (O_i_) with the help of holes, forming a positively charged defect (D_h_^2+^) at the site of the original O(―M)_n_: [30].

The energy barrier for oxygen vacancy migration via O_i_ for pure In_2_O_3_ is about 0.71 eV, which accounts for the requirement for the thermalization energy (certain temperature) to activate the V_O_s. Under negative gate bias, the conduction band tails are depleted of electrons, which suppresses Equation (3). However, as the bias is insufficient to pull the Fermi level down through the large number of D_e_ states, the localized valence band tail states can never be accumulated with holes, so Equation (2) is also suppressed. Only under light illumination combining with negative gate bias are holes in the valence band tail created, allowing the hole quasi-Fermi level to be pulled towards the valence band [30]. This permits Equation (2), and D_h_ states may be created at the expense of D_e_ states. Therefore, it is only the combination of photon illumination with energies sufficient to lead to hole generation in valence band tail states and certain temperature with thermalization energy higher than the energy barrier for oxygen vacancy migration as well as negative gate bias that will lead to the negative Δ*V_on_*, as observed experimentally.

### 3.7. Effect of the CT Transition of Pr^4+^/Tb^4+^ on the Photo Stability

**Figure 7:**
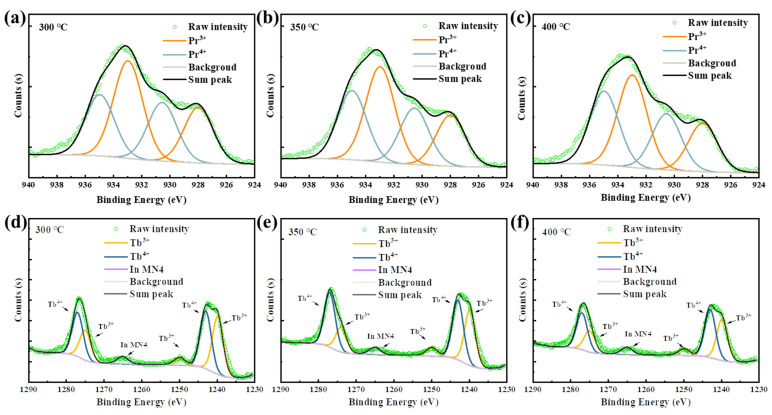
Pr 3*d* XPS spectra of the Pr:In_2_O_3_ films annealed at (**a**) 300, (**b**) 350, and (**c**) 400 °C; Tb 3*d* XPS spectra of the Tb:In_2_O_3_ films annealed at (**d**) 300, (**e**) 350, and (**f**) 400 °C.

The great improvement in NBIS stability of the Pr/Tb:In_2_O_3_ TFTs is ascribed to the charge transfer (CT) transition of the Pr^4+^ and Tb^4+^. It is known that tetravalent lanthanide ions (Ce^4+^, Pr^4+^ and Tb^4+^) have low CT transition energies that have broad-band absorption [31,32,33]. Compared to Ce^4+^, whose CT transition absorption is in the UV range, the CT transistor of Pr^4+^ and Tb^4+^ have broad absorption in the visible range [13,31]. To identify the existence of Pr^4+^/Tb^4+^ in the Pr/Tb:In_2_O_3_ films, X-ray photoelectron spectroscopy (XPS) experiments were performed. Figure 7 shows the Pr3*d* and Tb3*d* spectra of the Pr/Tb:In_2_O_3_ films. The peaks were fitted according to the analysis reported elsewhere [34,35]. The parameters of the fitting peaks for Pr3*d* and Tb3*d* are summarized in Tables S1 and S2, respectively. The ratio of Pr^4+^/Pr^3+^ or Tb^4+^/Tb^3+^ were calculated by the peak areas. There is a clear trend of Pr^4+^/Pr^3+^ < Tb^4+^/Tb^3+^ at the same annealing temperature. The higher content of Tb^4+^ in Tb:In_2_O_3_ compared to that of Pr^4+^ in Pr:In_2_O_3_ may be one of the reason for the higher mobility and stability of Tb:In_2_O_3_ TFTs compared to those of Pr:In_2_O_3_ TFTs because Tb^4+^ has a smaller radius with an isotropic half-full 4*f* ^7^ configuration and a quenched orbital moment. It should be noted that the 4*f* ^7^ configuration has a large magnetic moment that may scatter the carriers and decrease the mobility. It should be noted that the properties of the solution-processed Pr/Tb:In_2_O_3_ differed from those of the vacuum-processed ones greatly. We will not compare the difference between vacuum-processed Pr and Tb doped In_2_O_3_ TFTs this time. The more insightful mechanisms of the difference between Pr and Tb doped In_2_O_3_ is still under investigated and will be reported in the future.

Figure 6 illustrates the CT transition process in Pr^4+^/Tb^4+^ doped In_2_O_3_. Initially, the system is at Point A on Parabola (4*f* ^n^) corresponding with an electron at the top of the valence band and a Ln^4+^ ion (Ln4*f* ^n^—O2*p*^6^). Under light illumination, the initial state transfers to Point B of the Ln^3+^ state (Ln4*f* ^n+1^—O2*p*^5^). Due to Ln^3+^ having a larger ionic radius than Ln^4+^, it relaxes outward and moves to Point C. Then, it transfers back to Parabola (4*f* ^n^) via crossing relaxation (by thermal activation) where all luminescence is quenched [36,37,38]. It should be noted that the Ln4*f* ^n^—O2*p*^6^ to Ln4*f* ^n+1^—O2*p*^5^ CT transition has smaller overall lattice expansion/contraction and shorter relaxation time compared to V_O_ ionization because of the Coulomb attraction between the hole (O2*p*^5^) and the transferred electron [17,36]. As a result, the Ln^4+^ can more easily absorb light than V_O_. The results reveal that the improvement of the NBIS stability of the Pr/Tb:In_2_O_3_ TFTs is attributed to the absorption of the illuminated light by the Ln4*f* ^n^—O2*p*^6^ to Ln4*f* ^n+1^—O2*p*^5^ CT transition and downconversion of the light to nonradiative transition with a relatively short relaxation time compared to V_O_ ionization. Minasian et al. reported that the partially occupied 4*f* states are lower in energy than the unoccupied 5*d* states and provide band gaps of 2.3 and 1.7 eV for PrO_2_ and TbO_2_, respectively [39]. The lower energy level of Tb 4*f* ^7^ compared to Pr 4*f* ^1^ would make it easier for the Tb^4+^ to absorb the visible light than the Pr^4+^, which may be the main reason for the higher NBIS stability of Tb:In_2_O_3_ TFTs compared to Pr:In_2_O_3_ TFTs.

The positive Δ*V_on_* under NBIS at a low temperature (78 K, see Figure 5 and Figure S4) of the Pr/Tb:In_2_O_3_ TFTs was ascribed to the further trapping of electrons by the Ln4*f* ^n+1^ exciting states. At a low temperature, most of the electrons are localized in the tail states that intersect the Parabola (4*f* ^n+1^), as shown in Figure 6. Under negative gate bias, these localized electrons are depleted and a part of them can further transit to Point C via the Parabola (4*f* ^n+1^). As a result, the density of the localized electrons decreases, which is the reason why Pr/Tb:In_2_O_3_ TFTs exhibited positive Δ*V_on_* under NBIS at a low temperature.

### 3.8. Formatting of Mathematical Components

(1) $$I_{D}=\frac{W\mu C_{i}}{2L}{\left(V_{G}-V_{\mathrm{th}}\right)}^{2}$$

where *C*_i_ is the areal capacitance of the dielectric; *V*_th_ is the threshold voltage obtained by fitting the saturation region of *I_D_*^1/2^ versus *V_G_* plots and extrapolating the fitted line to *I_D_* = 0.

D_e_ + [O(—M)_n2_+2h] → D_e_+O_i_+D_h_^2+^ → O(—M)_n1_ + D_h_^2+^ (2)

D_h_^2+^ + [O(—M)_n1_+2e] → D_h_^2+^+ O_i_^2-^+ D_e_ → O(—M)_n2_ + D_e_ (3)

## 4. Conclusions

In conclusion, TFTs based on solution-processed In_2_O_3_ semiconductors doped with Pr^4+^ or Tb^4+^ were investigated. It was found that both Pr^4+^ and Tb^4+^ can improve the NBIS stability greatly, but Tb^4+^ is better than Pr^4+^. The undoped In_2_O_3_ TFTs with different annealing temperatures exhibited poor NBIS stability with serious turn-on voltage shift. As the annealing temperature increased, the Pr:In_2_O_3_ TFTs had poorer NBIS stability (Δ*V_on_* are −3.2, −4.8, and −4.8 V for annealing temperatures of 300, 350, and 400 °C, respectively), while the Tb:In_2_O_3_ TFTs had better NBIS stability (Δ*V_on_* are −3.6, −3.6, and −1.2 V for annealing temperatures of 300, 350, and 400 °C, respectively). When the measuring temperature decreased to 78 K, the pure In_2_O_3_ TFT showed little Δ*V_on_* after 3600 s NBIS, while the Pr:In_2_O_3_ and Tb:In_2_O_3_ TFTs exhibited positive Δ*V_on_* of +1.0 V and +0.8 V, respectively. Further studies reveal that the negative Δ*V_on_* under NBIS is the combining effect of photon illumination with energies sufficient to lead to hole generation in valence band tail states, a certain temperature with thermalization energy higher than energy barrier for oxygen vacancy migration, and negative gate bias that will lead to bending of the energy band. The improvement of the NBIS stability of the Pr^4+^/Tb^4+^:In_2_O_3_ TFTs is attributed to the absorption of the illuminated light by the Pr/Tb4*f* ^n^—O2*p*^6^ to Pr/Tb 4*f* ^n+1^—O2*p*^5^ charge transfer (CT) transition and downconversion of the light to nonradiative transition with a relatively short relaxation time compared to ionization of oxygen vacancies. The positive Δ*V_on_* of the Pr/Tb:In_2_O_3_ TFTs under low temperature (78 K) is attributed to the further trapping of electrons by the 4*f* ^n+1^ exciting states under negative bias. The higher NBIS stability of Tb:In_2_O_3_ TFTs compared to Pr:In_2_O_3_ TFTs is ascribed to the smaller ion radius of Tb^4+^ and the lower energy level of Tb 4*f* ^7^ compared to Pr 4*f* ^1^, which would make it easier for the Tb^4+^ to absorb the visible light than the Pr^4+^. It is worth noting that two sets of devices were tested, and the results can be considered typical.

## Figures and Tables

**Table 1:** Summary of the properties of the Pr/Tb:In_2_O_3_ TFTs with different annealing temperatures.

	*T* (°C)	*μ* (cm^2^·V^−1^·s^−1^)	*SS* (V/dec)	*V_th_* (V)	*I_on_/I_off_*	Δ*V_on_* under NBIS (V)
In_2_O_3_	300	16.6	0.27		5.6 × 10^7^	Bias dependent
	350	24.5	0.55		2.9 × 10^7^	Bias dependent
	400	23.4	0.68		6.8 × 10^6^	Bias dependent
Pr:In_2_O_3_	300	5.0	0.11	1.8	8.4 × 10^9^	−3.2
	350	6.1	0.14	1.5	3.0 × 10^7^	−4.8
	400	10.1	0.13	0.4	5.1 × 10^7^	−4.8
Tb:In_2_O_3_	300	4.7	0.12	2.6	3.7 × 10^9^	−3.6
	350	13.4	0.17	1.2	1.8 × 10^8^	−3.6
	400	18.2	0.15	1.4	6.9 × 10^7^	−1.2

## Data Availability

The data is available on reasonable request from the corresponding author.

## Supplementary Materials

The following supporting information can be downloaded at: https://www.mdpi.com/article/10.3390/nano12213902/s1, Figure S1: XRD patterns of the Pr/Tb:In_2_O_3_ films annealed at different temperatures, and the bottom pattern is the standard refraction pattern of the single-crystal bixbyite In_2_O_3_; Figure S2: The variations of time-dependent transfer curves under NBIS (a negative gate bias stress of −30 V combining with white LED light illumination of 250 Lux) for the TFTs with channels of In_2_O_3_ annealed at (a) 300, (b) 350, and (c) 400 °C; Figure S3: The variations of time-dependent transfer curves under NBIS (a negative gate bias stress of −30 V combining with white LED light illumination of 250 Lux) for TFTs with channels of (a) Pr:In_2_O_3_ and (b) Tb:In_2_O_3_ annealed at 400 °C; Figure S4: Variations of time-dependent transfer curves under NBIS with different temperatures of 78 K, 150 K, and 220 K (a negative gate bias stress of −20 V combining with white LED light illumination of 250 Lux) for TFTs with channels of In_2_O_3_ (measured under (a) 78 K, (b)150 K, (c) 220 K), Pr:In_2_O_3_ (measured under (d) 78 K, (e)150 K, (f) 220 K), and Tb:In_2_O_3_ (measured under (g) 78 K, (h)150 K, (i) 220 K); Figure S5: Variations of time-dependent transfer curves under NBS without light illumination at room temperature for the TFTs with channels of (a) In_2_O_3_, (b) Pr:In_2_O_3_, and (c) Tb:In_2_O_3_; Table S1: Summary of the parameters of the fitting peaks of the XPS Pr 3*d* spectra in Figure 7; Table S2: Summary of the parameters of the fitting peaks of the XPS Tb 3*d* spectra in Figure 7.
