# Supplementary material for: The Mechanism of the Photostability Enhancement of Thin-Film Transistors Based on Solution-Processed Oxide Semiconductors Doped with Tetravalent Lanthanides
Source: Nanomaterials (Basel). 2022 Nov 4;12(21):3902. doi: 10.3390/nano12213902 (PMC9655621; doi:10.3390/nano12213902)
Supplement: Supplementary file 1 [file nanomaterials-12-03902-s001.zip › nanomaterials-1981583-supplementary conversion.pdf]

nanomaterials

Supplementary Information

The Mechanism of the Photostability Enhancement of Thin-Film Transistors Based on Solution-Processed Oxide Semiconductors Doped with Tetravalent Lanthanides

Linfeng Lan [1,*], Chunchun Ding [1], Penghui He [2], Huimin Su [1], Bo Huang [1], Jintao Xu [1], Shuguang Zhang [1] and Junbiao Peng [1]

[1] State Key Laboratory of Luminescent Materials and Devices, South China University of Technology, Guangzhou 510640, China
[2] School of Physics and Electronics, Hunan University, Changsha 410082, China
* Correspondence: lanlinfeng@scut.edu.cn;

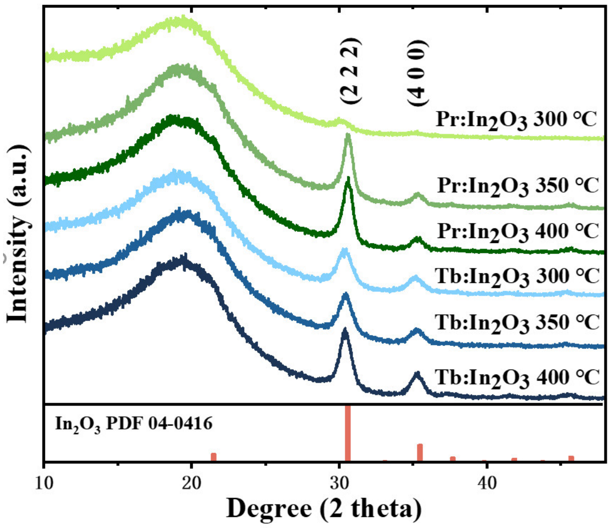

Figure S1. XRD patterns of the Pr/Tb:In_2O_3 films annealed at different temperatures, and the bottom pattern is the standard refraction pattern of the single-crystal bixbyite In_2O_3.

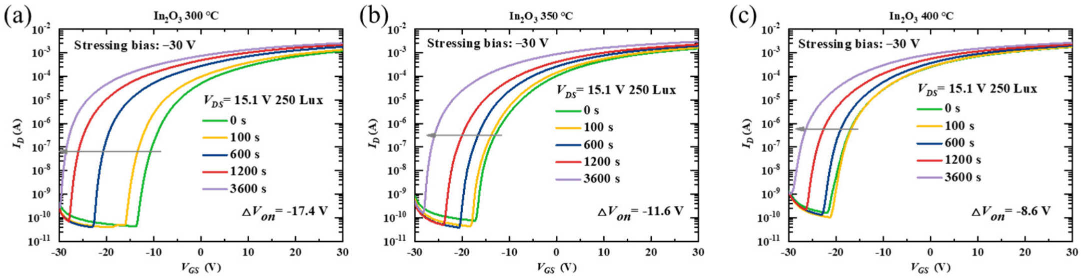

Figure S2. The variations of time-dependent transfer curves under NBIS (a negative gate bias stress of −30 V combining with white LED light illumination of 250 Lux) for the TFTs with channels of In_2O_3 annealed at (a) 300, (b) 350, and (c) 400 °C.

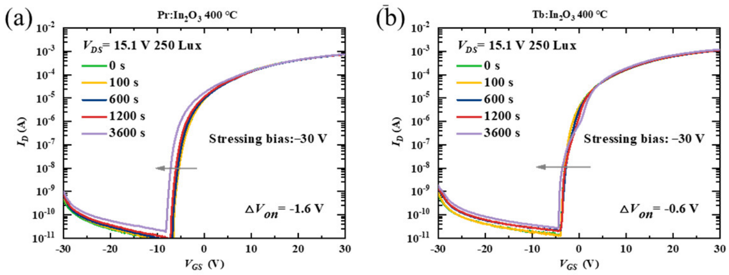

Figure S3. The variations of time-dependent transfer curves under NBIS (a negative gate bias stress of −30 V combining with white LED light illumination of 250 Lux) for the TFTs with channels of (a) Pr:In_2O_3 and (b) Tb:In_2O_3 annealed at 400 °C.

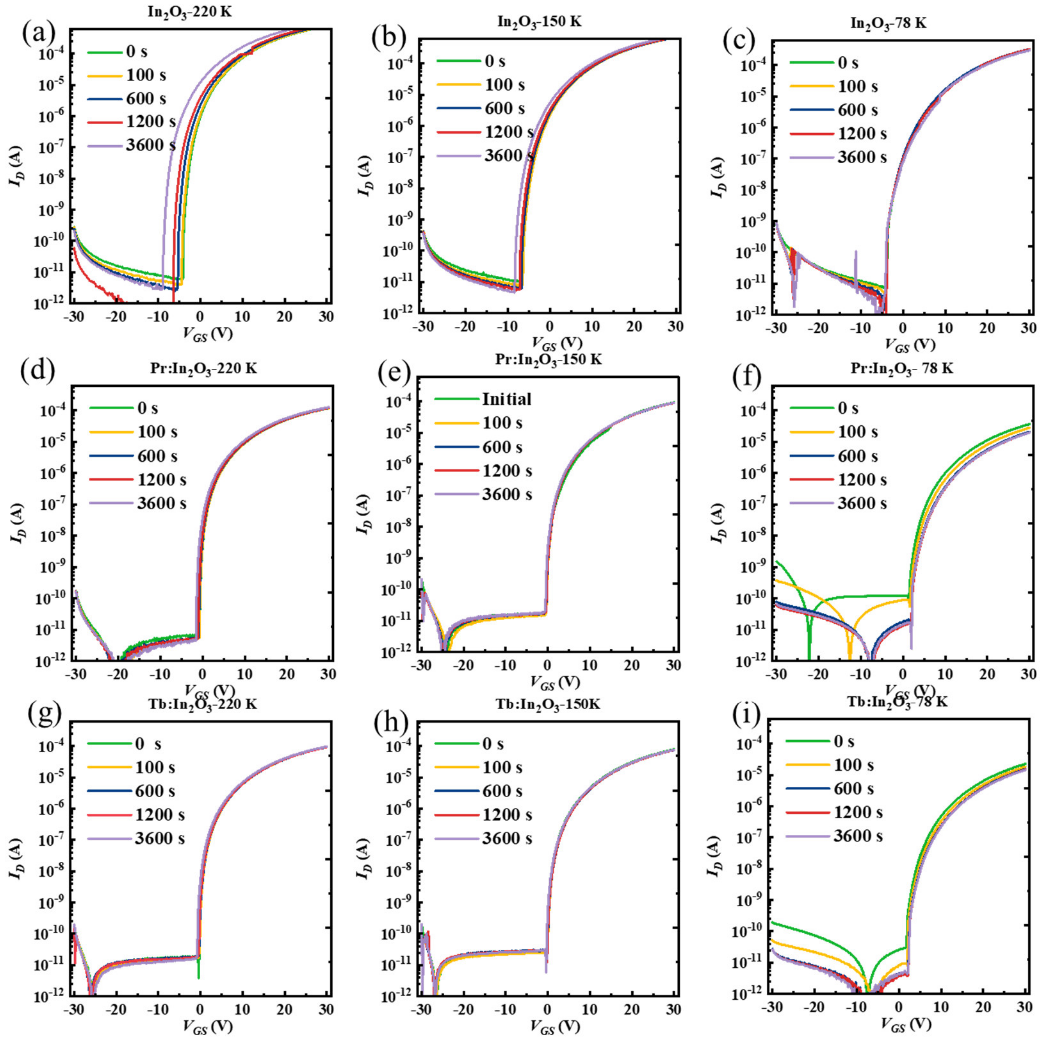

Figure S4. Variations of time-dependent transfer curves under NBIS with different temperatures of 78 K, 150 K, and 220 K (a negative gate bias stress of −20 V combining with white LED light illumination of 250 Lux) for the TFTs with channels of In_2O_3 (measured under (a) 78 K, (b)150 K, (c) 220 K), Pr:In_2O_3 (measured under (d) 78 K, (e)150 K, (f) 220 K), and Tb:In_2O_3 (measured under (g) 78 K, (h)150 K, (i) 220 K).

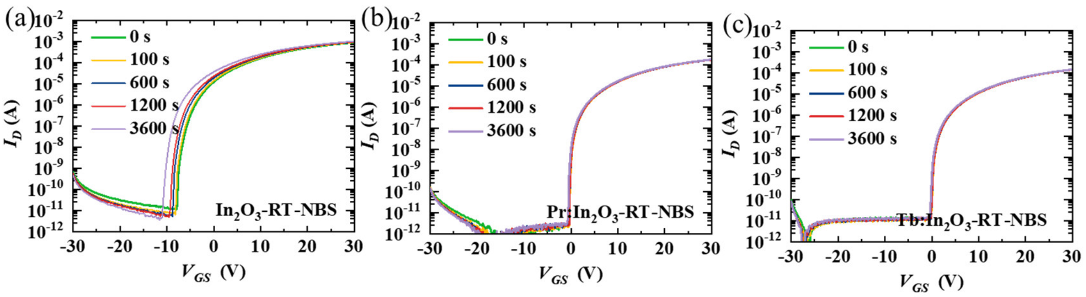

Figure S5. Variations of time-dependent transfer curves under NBS without light illumination at room temperature for the TFTs with channels of (a) In_2O_3, (b) Pr:In_2O_3, and (c) Tb:In_2O_3.

Table S1. Summary of the parameters of the fitting peaks of the XPS Pr 3*d* spectra in Figure 7.

**Temperature (°C)**	**Pr 3$d_{5/2}$ Binding Energy (eV)**				**Pr Composition (%)**	
	928	931	933	935	Pr^{3+}	Pr^{4+}
300	17.38	19.69	36.79	26.14	54.17	45.83
350	16.68	20.69	35.15	25.48	51.83	48.17
400	17.83	20.69	34.17	27.31	52.00	48.00

Table S2. Summary of the parameters of the fitting peaks of the XPS Tb 3*d* spectra in Figure 7.

**Temperature (°C)**	**Tb 3$d_{5/2}$ Binding Energy (eV)**			**Tb Composition (%)**	
	1239.8	1243	1249.8	Tb^{3+}	Tb^{4+}
300	22.87	23.00	3.07	53.00	47.00
350	21.99	23.79	3.41	51.64	48.36
400	23.76	27.81	3.86	49.83	50.17
